# Unveiling the Synaptic Function and Structure Using Paired Recordings From Synaptically Coupled Neurons

**DOI:** 10.3389/fnsyn.2020.00005

**Published:** 2020-02-11

**Authors:** Guanxiao Qi, Danqing Yang, Chao Ding, Dirk Feldmeyer

**Affiliations:** ^1^Institute of Neuroscience and Medicine, INM-10, Jülich Research Centre, Jülich, Germany; ^2^Department of Psychiatry, Psychotherapy and Psychosomatics, RWTH Aachen University Hospital, Aachen, Germany; ^3^Jülich-Aachen Research Alliance, Translational Brain Medicine (JARA Brain), Aachen, Germany

**Keywords:** paired recordings, synaptic connection, structure-function analysis, quantal analysis, neuromodulation

## Abstract

Synaptic transmission between neurons is the basic mechanism for information processing in cortical microcircuits. To date, paired recording from synaptically coupled neurons is the most widely used method which allows a detailed functional characterization of unitary synaptic transmission at the cellular and synaptic level in combination with a structural characterization of both pre- and postsynaptic neurons at the light and electron microscopic level. In this review, we will summarize the many applications of paired recordings to investigate synaptic function and structure. Paired recordings have been used to study the detailed electrophysiological and anatomical properties of synaptically coupled cell pairs within a synaptic microcircuit; this is critical in order to understand the connectivity rules and dynamic properties of synaptic transmission. Paired recordings can also be adopted for quantal analysis of an identified synaptic connection and to study the regulation of synaptic transmission by neuromodulators such as acetylcholine, the monoamines, neuropeptides, and adenosine etc. Taken together, paired recordings from synaptically coupled neurons will remain a very useful approach for a detailed characterization of synaptic transmission not only in the rodent brain but also that of other species including humans.

## Introduction

To understand local neuronal microcircuits in the brain, it is necessary to know the morphological and electrophysiological properties of both the pre- and postsynaptic neurons, the synaptic connection type(s) and their structure-function relationship. However, in many studies of synaptic transmission the identity of the pre- and postsynaptic neuron is not well or not at all characterized. This is because of the relatively unspecific stimulation protocols (e.g., extracellular stimulation) often used to investigate synaptic connectivity, which generally do not allow to determine the structural and functional properties of the presynaptic neuron. Paired recordings together with intracellular staining by markers such as biocytin/neurobiotin and/or fluorescent dyes are better suited for studying local neuronal microcircuits. This technique permits a simultaneous, correlated characterization of the structural and functional properties of a synaptic connection.

Monosynaptic connections between identified neurons have been investigated in both cortical and subcortical brain regions using paired recordings in acute brain slices (Malinow, 1991; Mason et al., 1991; Buhl et al., 1994; Deuchars et al., 1994; Bolshakov and Siegelbaum, 1995; Miles et al., 1996; Stratford et al., 1996; Geiger et al., 1997; Markram et al., 1997a; Thomson and Deuchars, 1997; Feldmeyer et al., 1999; Gupta et al., 2000; Tamas et al., 2000, 2003; Holmgren et al., 2003; Szabadics et al., 2006; Helmstaedter et al., 2008; Olah et al., 2009, for reviews, see Miles and Poncer, 1996; Debanne et al., 2008; Feldmeyer and Radnikow, 2016). Sharp microelectrodes were initially used in these experiments (Mason et al., 1991; Buhl et al., 1994; Deuchars et al., 1994). However, electrophysiological recordings with sharp microelectrodes have several limitations, e.g., the electrical noise is high and the membrane seal poor, the approach is generally blind and thus the inter-somatic distance between pre- and postsynaptic neurons not well controlled (Brette and Destexhe, 2012). Later, patch pipettes were employed in order to measure synaptic responses with a higher signal-to-noise ratio and an improved temporal resolution. A significant advance was the use of infrared differential interference contrast optics (Dodt and Zieglgansberger, 1990) that significantly improved the visual identification of neurons in acute brain slices (Stuart et al., 1993) so that it became possible to obtain recordings from synaptic connections between visually identified neurons.

An advantage of paired recordings is the fact that functional characterization can be combined with the morphological and/or molecular analysis at both the light and electron microscopic level (Deuchars et al., 1994; Markram et al., 1997a, 1998b; Reyes et al., 1998; Feldmeyer et al., 2002, 2006; Silver et al., 2003; Tamas et al., 2003; Kapfer et al., 2007; Silberberg and Markram, 2007; Helmstaedter et al., 2008). After histochemical processing, the expression of specific marker proteins of the synaptically connected neuron pair can be determined, in a subsequent step the somatodendritic and axonal morphologies recovered and then reconstructed in three spatial dimensions. This will allow a quantitative analysis of morphological features such as orientation, branching pattern, spatial length density etc. These parameters could provide a basis for an objective classification of pre- and postsynaptic neurons in a specific synaptic connection. Furthermore, paired recordings also permit the identification of synaptic contacts of unitary synaptic connections using a combination of light and electron microscopy. In addition to this detailed analysis of the synaptic transmission at a defined neuronal microcircuit paired recordings also allow the study of quantal properties of identified synapses and the modulation of synaptic transmission by neurotransmitters such as acetylcholine, noradrenaline, dopamine, serotonin, and adenosine.

## Electrophysiological, Morphological and/or, Molecular Characterization of Synaptic Connections in Local Neuronal Microcircuits

The most crucial step for paired recordings in acute brain slices is to find a sufficiently stable synaptic connection so that a detailed analysis of its structural and functional properties is possible. This step depends on several important factors which will be discussed here in brief (for more details, see Radnikow et al., 2012; Feldmeyer and Radnikow, 2016). First, it is important to determine the optimal procedure for preparing brain slices so that the axo-dendritic branches of both pre- and postsynaptic neuron for the synaptic connection under study is well preserved. A suitable slice thickness needs to be determined depending on the recording configuration (whole-cell with patch pipettes or intracellular with sharp microelectrodes); an increase in the slice thickness may significantly increase the connection probability and the quantification of synapse number per connection (Thomson and Lamy, 2007; Stepanyants et al., 2009). Second, the composition of solutions used during the slicing and incubation needs to be adjusted carefully according to the age of animals and type of species. Several slicing and incubation solutions for adult and senescent animal and human brain tissue are available under http://www.brainslicemethods.com/ (Ting et al., 2014, 2018a,b). Finally, the connection probability of different neuron types is highly variable (from 5 to 70%) depending on both the presynaptic axonal projection and the postsynaptic dendritic arborization (Thomson and Lamy, 2007; Lefort et al., 2009; Fino et al., 2013; Pfeffer et al., 2013; Jiang et al., 2015; Markram et al., 2015; Radnikow et al., 2015; Seeman et al., 2018; Jouhanneau and Poulet, 2019). Therefore, choosing the appropriate strategy, either a random patch or a “searching” protocol (Qi et al., 2015), is critical for the success of paired recordings. Paired recordings from synaptically coupled neurons allow a wide variety of functional and structural analysis. The most relevant issues will be described below.

### Electrophysiological Characterization of Local Synaptic Transmission

**Figure 1:**
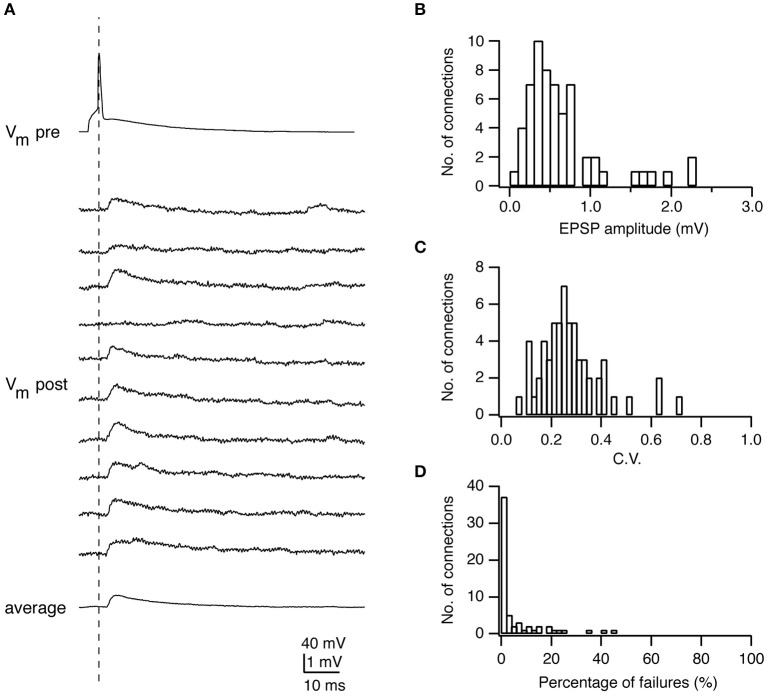
Electrophysiological characterization of synaptic connections using paired recordings. **(A)** Paired recordings from a synaptic connection established between a presynaptic L4 spiny neuron and a postsynaptic L2/3 pyramidal cell. Top, a presynaptic AP; Middle, ten successive EPSPs in response to a presynaptic AP; Bottom, the average EPSP. **(B)** Histogram of the EPSP amplitudes in L4-L2/3 connections (*n* = 64). **(C)** Histogram of the coefficients of variation (C.V.s) of EPSPs in L4-L2/3 connections (*n* = 64). **(D)** Histogram of the failure rate (in %) in L4-L2/3 connections (*n* = 64). Figure has been adapted from Feldmeyer et al. (2002) with permission.

The synaptic strength (or weight) is a key parameter to characterize the efficacy of a synaptic connection. It reflects whether the synaptic connection has a strong or weak influence on postsynaptic output. It is measured as the peak amplitude of postsynaptic potentials (PSPs) evoked by presynaptic action potentials (APs). For excitatory synaptic connections in the neocortex, the PSP amplitude is not normally distributed but skewed toward lower values (~0.5 mV) with a long tail with higher values (>2 mV) (Figures 1A,B) (Markram et al., 1997a; Feldmeyer et al., 1999, 2002, 2006; Sjostrom et al., 2001; Holmgren et al., 2003; Lefort et al., 2009). It has been shown by theoretical analysis that this synaptic weight distribution can be understood through optimization of information storage in neuronal networks (Brunel et al., 2004; Varshney et al., 2006; Barbour et al., 2007). It has also been suggested that the high-amplitude connections represent rare, strong connections that mediate stimulus-specific response amplification in cortical microcircuits (Cossell et al., 2015).

The time course of postsynaptic response is another important determinant of the computational power of a synaptic connection and significantly affects the synaptic integration in postsynaptic neurons. Long-lasting PSPs show a stronger summation while brief postsynaptic responses are necessary to achieve a high temporal fidelity for repetitive synaptic inputs. Quantitatively, the time course of excitatory or inhibitory PSPs (EPSPs/IPSPs) is described by its 20–80% rise time, decay time constant and half-width. It should be noted that the EPSP/IPSP time course is shaped by (low-pass) dendritic filtering due to the distance between the recording site (normally at the soma) and the synapse location (Rall, 1967).

The latency is defined as the time difference between the peak of presynaptic AP and the beginning of the PSP. The size and variation of latencies determines the time window of integration of the synaptic response. Many factors such as the fine structure of the pre- and postsynaptic sites, the release probability of neurotransmitters, and the passive and/or active electrophysiological properties of both pre- and postsynaptic neurons affect the latency in synaptic transmission.

The reliability is an important property of a synaptic connection, which characterizes the extent of the PSP variability. Synaptic reliability and variability are sensitive to recording conditions, e.g., the temperature and Ca^2+^ concentration in the recording solution. The reliability of synaptic transmission increases with the increasing temperature (Hardingham and Larkman, 1998; Volgushev et al., 2004) and Ca^2+^ concentration (Rozov et al., 2001; Silver et al., 2003) due to enhanced transmitter release. To determine this parameter, an AP is elicited in the presynaptic neuron resulting in an EPSP or IPSP in the postsynaptic neuron (Figures 1A, 4A). Between 50 and 100 sweeps are recorded to determine the mean amplitude of the synaptic response (Figures 1B, 4B) and its variance. A frequently used measure for the reliability is the coefficient of variation (CV) which is defined as:

$$\begin{array}{l} {\text{CV}}_{\text{PSP}}=\surd \left(\sigma_{\text{PSP}}^{2}-\sigma_{\text{Noise}}^{2}\right)/\mu_{\text{PSP}} \end{array}$$

where $\sigma_{\text{PSP}}^{2}$ is the variance of the PSP amplitude, $\sigma_{\text{Noise}}^{2}$ the variance of the membrane potential fluctuation, and μ_PSP_ the mean PSP amplitude (Figure 1C). The variance of the PSP is corrected by subtracting the membrane potential variance, which includes membrane potential noise (i.e., from random ion channel openings) and electrical noise introduced by the recording equipment. CV_PSP_ is a surrogate measure for the release probability of transmitters. However, this measure is only indirect and a detailed quantal analysis (see below) is needed to determine its actual value.

The failure rate is defined as the frequency with which a synapse fails to respond to a presynaptic AP (Figure 1D). In general, synaptic connections with a low neurotransmitter release probability (e.g., synapses formed by L6A cortico-thalamic pyramidal neurons) (Yang et al., 2019) and/or few synaptic contacts (e.g., synapse formed between parallel fibers from granule cells and Purkinje cell dendrites) (Isope and Barbour, 2002) show a significant number of failures. However, failures may not be apparent despite a relatively low release probability when the number of synaptic contacts is sufficiently large. Under this condition it is likely that vesicle release would occur at least at a small fraction of synaptic contacts; hence, no failures would be observed. This is in accordance with findings in a number of paired recording studies in acute cortical slices that generally report a low failure rate of synaptic transmission (Atzori et al., 2001; Koester and Johnston, 2005; Feldmeyer et al., 2006; Frick et al., 2008; Lefort et al., 2009).

Changes in the strength of the synaptic response are critical for the flexibility and plasticity of synaptic function. For monosynaptic connections, paired recordings have shown that, during the delivery of multiple stimuli at short time intervals, the size of the postsynaptic responses can become either larger or smaller, a phenomenon known as short-term facilitation or depression, respectively. When the release probability is low during the initial presynaptic AP, PSP facilitation is likely to occur. This is likely to results from an increase in the Ca^2+^ concentration in the presynaptic terminal with each successive presynaptic AP which will lead to successively larger PSPs (i.e., an increase in release probability). After some time the release probability and hence the PSP amplitude will decrease again because of a depletion of the readily releasable pool of synaptic vesicles (see below). Short-term synaptic depression, on the other hand, occurs when the initial release probability is high, i.e., when many synaptic vesicles are released during the first presynaptic AP. This then results in a transient depletion of synaptic vesicle from the readily releasable pool (Zucker and Regehr, 2002; Rizzoli and Betz, 2004, 2005). Whether a synaptic connection shows short-term facilitation or depression depends on the pre- and/or postsynaptic neuron identity (Markram et al., 1998b; Reyes et al., 1998; Scanziani et al., 1998; Gupta et al., 2000; Koester and Johnston, 2005; Ma et al., 2012) (Figure 3). By eliciting a pair (or train) of APs in the presynaptic neuron at a fixed interval (e.g., 100 ms) and measuring the amplitude of the postsynaptic response, the paired-pulse ratio (PPR) is calculated as PPR = PSP_2_/PSP_1_. The PPR is commonly used to characterize short-term synaptic plasticity and specifies whether the initial release probability is high or low. Although the PPR is widely used, it is not sufficient to unmask the interplay between release, depression and facilitation (Dittman et al., 2000). There is some ambiguity in using the PPR to determine depression/facilitation dynamics in the case of strongly facilitating synapses. In these synapses, PPR might be small for the first two PSPs and gradually becomes larger during repetitive presynaptic stimulation (Markram et al., 1998a). For such cases a train of frequency-dependent APs elicited in the presynaptic neuron is more appropriate to be adopted for measuring the postsynaptic response.

Synaptic function is also affected by retrograde messengers (e.g., glutamate, GABA, endocannabinoid) released from postsynaptic dendrites (Zilberter et al., 2005). Paired recordings between layer 2/3 pyramidal cells and bitufted interneurons showed that the dendritic GABA release depresses excitatory transmission via presynaptic metabotropic GABA_B_ receptors in the rat neocortex (Zilberter et al., 1999). For the inhibitory transmission, depolarization-induced suppression of inhibition (DSI) was found widely in different cortical areas including the hippocampus (Wilson and Nicoll, 2001), cerebellum (Kreitzer and Regehr, 2001), and neocortex (Trettel and Levine, 2003). DSI has been shown to be caused by the postsynaptic deporalization-induced dendritic release of endocannabinoids, which diffuse retrogradely to presynaptic axonal terminals where they bind to cannabinoid 1 receptors to reduce the GABA release.

It should be noted that there are some differences between *in vitro acute brain slice (or ex vivo)* and *in vivo* recording conditions. Therefore, the property of synaptic transmission studied *in vitro* may be different from that *in vivo* condition. A prominent difference is the extracellular Ca^2+^ concentration which is ~1.2–1.3 mM free Ca^2+^ in the cerebrospinal fluid (Heinemann et al., 1977; Massimini and Amzica, 2001; Crochet et al., 2005; Borst, 2010) but 2 mM Ca^2+^ compound in a standard extracellular perfusion solution. Because calcium salts do not fully dissociate the free Ca^2+^ concentration in the extracellular fluid will be lower than the absolute CaCl_2_ concentration [or any other calcium salt this is substituted for CaCl_2_ (e.g., Ca(CH_3_SO_3_)_2_)]. An absolute CaCl_2_ concentration of 2 mM amounts to 1.7 mM free Ca^2+^ (as can be measured with an ion-selective electrode and/or calculated from the dissociation constant). Thus, compared to the *in vitro* condition, the PSP amplitude and reliability will be lower and the failure rate higher under *in vivo* condition because of the reduced synaptic release probability. In addition, the short-term synaptic plasticity is likely to change from strong depression to no change or weak facilitation. In addition, the membrane conductance of neocortical neurons is high *in vivo* because of the intense synaptic bombardment, which rarely appears under *in vitro* conditions (Destexhe et al., 2003). Therefore, the time course of PSPs recorded *in vivo* is also different from that *in vitro*, e.g., the decay of PSPs is faster *in vivo* than *in vitro* because of enhanced membrane conductances.

Long-term synaptic changes such as long-term potentiation (LTP) and depression (LTD) have been considered as the cellular mechanism of learning and memory (Huganir and Nicoll, 2013). Paired recordings have been widely adopted to investigate the LTP and LTD and uncover their induction conditions and mechanisms (Malinow, 1991; Arancio et al., 1995; Bolshakov and Siegelbaum, 1995; Liao et al., 1995; Markram et al., 1997b; Bi and Poo, 1998; Egger et al., 1999; Montgomery et al., 2001). For example, the postsynaptic insertion of AMPA receptors has been considered to be the molecular basis of LTP induction. Spike-timing-dependent plasticity (STDP) is one Hebbian type of long-term synaptic plasticity. Its induction depends on the precise timing of pre- and postsynaptic AP firing. Paired recordings between layer 5 pyramidal cells showed that if a presynaptic neuron fires earlier (e.g., +10 ms) than its postsynaptic neuron, LTP will be induced. Otherwise, if the presynaptic neuron fires later (e.g., −10 ms) than its postsynaptic neuron, LTD will develop (Markram et al., 1997b; Bi and Poo, 1998, 2001; Abbott and Nelson, 2000). However, this rule does not apply to synaptic connections established between layer 4 spiny neurons. Whether presynaptic neurons fire earlier or later (e.g., ±10 ms) than postsynaptic neurons LTD will always be induced because of presynaptic metabotropic glutamate receptor activation (Egger et al., 1999).

In addition to chemical synapses, synaptic coupling can also occur via electrical synapses or gap junctions, in particular between immature neurons and interneurons of the same type. Paired recordings are also feasible to record from neurons coupled via gap junctions and to characterize their electrical properties such as the coupling coefficient and junctional conductance (Galarreta and Hestrin, 1999; Gibson et al., 1999). When combining with the biocytin labeling, the morphological properties of gap junctions can be studied at both light and electron microscopic levels as described below (Tamas et al., 2000).

Paired (or multiple) recordings allow to study the organization principles of neuronal networks and shed light on their fundamental features. Previous connectivity studies suggest that neuronal networks are not randomly connected but may have a fine-scale specificity of connectivity (Song et al., 2005; Brown and Hestrin, 2009; Yu et al., 2009; Ko et al., 2011; Perin et al., 2011; Jiang et al., 2013; Cossell et al., 2015). For example, it was demonstrated that two excitatory neurons are more likely to be connected if they share a common neighbor, the so-called “common neighbor rule,” in neuronal networks of cortical layers 2/3 and 5 (Song et al., 2005; Ko et al., 2011; Perin et al., 2011). The preference of connection formation between two excitatory neurons also depends on their long-range axonal targets (Brown and Hestrin, 2009), developing origins (Yu et al., 2009) and orientation selectivities (Ko et al., 2011).

### Morphological and/or Molecular Characterization of Synaptic Connections

For a detailed characterization of the morphological properties of synaptic connections, an optimal biocytin filling and a careful histochemical processing are of major importance. We have optimized these procedures in our laboratory (see Marx et al., 2012; Radnikow et al., 2012; Qi et al., 2015; Feldmeyer and Radnikow, 2016).

**Figure 2:**
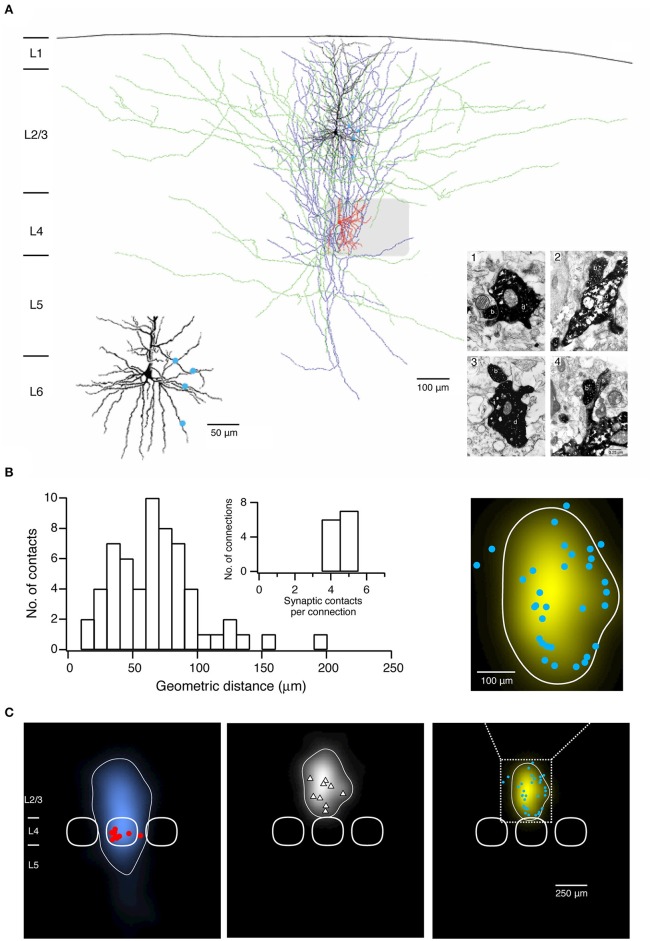
Morphological characterization of synaptic connections using paired recordings in combination with biocytin fillings. **(A)** Morphological reconstruction of a synaptically coupled cell pair between a L4 spiny stellate cell and a L2/3 pyramidal neuron. The somatodendritic and axonal compartments of the presynaptic spiny stellate cell are drawn in red and blue, respectively. The somatodendritic and axonal compartments of the postsynaptic L2/3 pyramidal neuron are drawn in black and green, respectively. The gray square represents the L4 barrel where the spiny stellate cell is located. Left inset, four putative synaptic contacts established by the axon of the L4 spiny stellate cell with the dendrites of the L2/3 pyramidal neuron are marked by blue dots. Right inset, electron micrographs of the synaptic contacts. All four synaptic contacts which were identified with the light microscope were confirmed at the electron microscopic level. The axonal boutons (b) of the L4 spiny stellate cell established synaptic contacts on dendritic shafts (d) in contacts 1–3 while on a dendritic spine in contact 4 of the L2/3 pyramidal neuron. **(B)** Histogram of the geometric distances from the somata of putative synaptic contacts in 13 L4 spiny neuron-L2/3 pyramidal cell pairs. Inset, distribution of number of synaptic contacts per connection. **(C)** 2D maps of axonal (left) and dendritic (middle) “length density” of synaptically coupled L4 spiny neurons and L2/3 pyramidal cells (*n* = 9), aligned with respect to the barrel center. The predicted innervation domain (right) of L2/3 dendrites by L4 axons is given by the product of the L4 axonal density and the L2/3 dendritic density. Contours (thin lines) enclosing 80% of the integrated density are superimposed. Positions of L4 spiny neuron sonata (red dots), L2/3 pyramidal cell sonata (white triangles), putative synaptic contacts (cyan dots), and outlines of barrels (thick lines) are indicated symbolically. Inset, zoom in the predicted innervation domain superimposed by putative synaptic contacts. **(A,B)** have been adapted from Feldmeyer et al. (2002) with permission and **(C)** from Lubke et al. (2003) with permission.

Following histochemical processing biocytin-labeled neuronal cell pairs are inspected under the light microscope using a 100× or a 50× oil immersion objective. Oil immersion objectives with a high numerical aperture (= 1.4) have to be used in order to focus throughout the entire slice thickness (~300 μm). Computer-assisted 3D neuronal reconstructions are made using the Neurolucida® system (Microbrightfield). This is a neuroanatomical reconstruction system for tracing the neuronal somatodendritic and axonal branches in all three dimensions (3D). Tracing is normally done manually; automatic or semi-automatic tracing approaches are often not applicable because of the dense and profuse branching of the dendritic branches and in particular axonal collaterals of the pre- and postsynaptic neurons (Figure 2A). Dendrites and axons are traced at high resolution, i.e., with 0.5–1.0 μm step size in z-direction. Furthermore, frequent alignments in the *x, y*, and *z*-dimensions of the neurons are required.

To identify synaptic contacts formed between the pre- and postsynaptic neurons a light microscope with the highest magnification [e.g., 1000×, 100× objective (oil immersion) and 10× eyepiece] is used. Putative synaptic contacts are defined as locations where a presynaptic axonal bouton comes near or overlaps with a dendritic spine or shaft of the postsynaptic neuron at the same focus (Figure 2A). Then, the spatial distribution of putative synaptic contacts on postsynaptic somatodendritic compartments can be determined (Figure 2B). In order to verify putative synaptic contacts identified under a light microscope a subsequent electron microscopic (EM) analysis is required (Markram et al., 1997a; Feldmeyer et al., 2002); under EM pre- and postsynaptic axonal boutons and dendritic spines or shafts, respectively, can be identified unambiguously (Figure 2A).

A quantitative morphological analysis of reconstructed neurons can be performed using the Neuroexplorer® (Microbrightfield) software. This software extracts parameters including the length of axonal and dendritic branches, the degree of arborization, the orientation etc., which can be used to classify neuronal cell types, e.g., by using the cluster analysis. Furthermore, morphological data about the axonal and dendritic arborization of the pre- and postsynaptic neurons can be further processed to calculate axonal and dendritic length 'density maps' (Figure 2C) (Lubke et al., 2003; Narayanan et al., 2015). These “density maps” could reflect a general pattern of axonal or dendritic length distribution across the layers and columns. By calculating the product of the presynaptic axonal density with the postsynaptic dendritic density, the average 'innervation domains' can be determined (Figure 2C). Such 'innervation domains' delineate the probability distribution of synaptic contacts for an identified synaptic microcircuit (Lubke et al., 2003; Stepanyants and Chklovskii, 2005).

**Figure 3:**
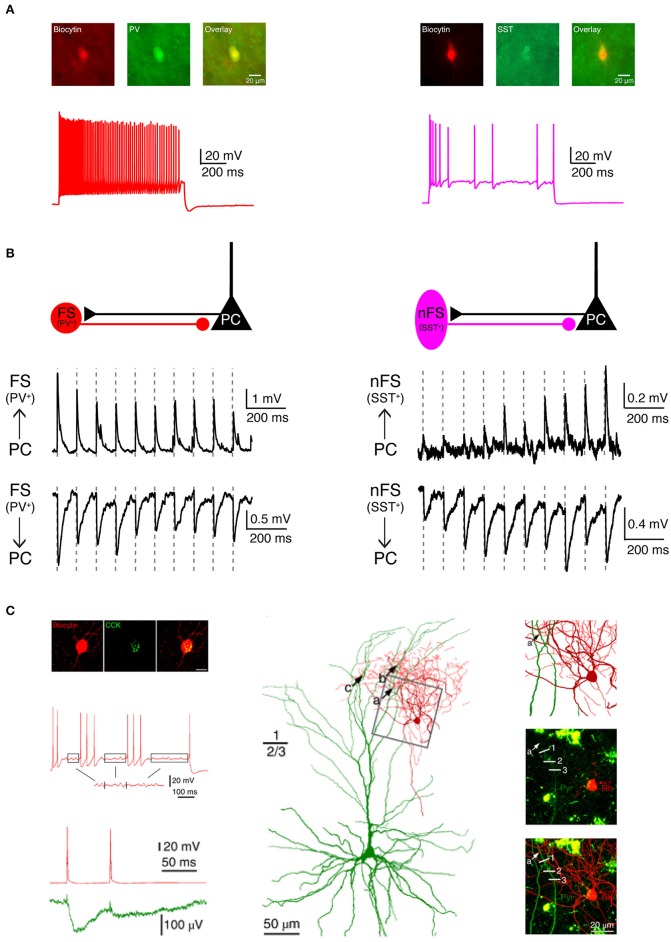
Electrophysiological, morphological and molecular characterization of synaptic connections by combining paired recordings with immuno-fluorescent stainings for specific marker proteins. **(A)** Two main types of GABAergic interneurons in the neocortex are PV^+^ fast spiking interneurons (left, red) which express the Ca^2+^-binding protein parvalbumin (PV) and SST^+^ non-fast spiking interneurons (right, violet) which express the neuropeptide somatostatin (SST). **(B)** Two interneuron types form synaptic connections with different characteristics. Left, PV^+^ fast spiking interneurons receive initially strong but quickly depressing EPSPs from neighboring excitatory neurons. At the same time, they produce depressing IPSPs in synaptically connected neighboring excitatory neurons. Right, SST^+^ non-fast spiking interneurons, in contrast, receive initially weak and gradually facilitating EPSPs from neighboring excitatory neurons and in turn elicit facilitating IPSPs in their target excitatory neurons. **(C)** Boldog et al. identified a specialized human cortical GABAergic cell type, the so-called L1 rosehip cell (RC). L1 RCs express cholecystokinin (CCK), but not PV, SST, or other molecular markers. L1 RCs exhibit an intermittent non-fast spiking firing pattern with subthreshold membrane potential oscillations (boxed segments). By combining paired recordings with Ca^2+^ imaging the authors were able to demonstrate that L1 RCs establish inhibitory synapses onto apical dendritic tufts of L2/3 pyramidal cells to regulate the AP backpropagation in a segment-specific manner. Electrical signals and morphologies of L1 RCs are in red and those of L2/3 pyramidal cells in green. **(A,B)** have been adapted from Feldmeyer et al. (2018) with permission and **(C)** from Boldog et al. (2018) with permission.

In addition to biocytin labeling alone, a combination with immunofluorescent staining is also possible, e.g., for specific molecular marker proteins such as Ca^2+^-binding protein/neuropeptide like parvalbumin, somatostatin, vasoactive intestinal polypeptide (VIP), cholecystokinin (CCK) or transcription factor like Fez2, CTIP2, Foxp2 for different inhibitory and excitatory neuron types, respectively (Figures 3A,C). For this, the neuron is filled with biocytin and a biocytin-conjugated fluorescent dye during the electrophysiological recording (e.g., Alexa Fluor 594) so that it is easily distinguished from other neurons after paraformaldehyde fixation. In a second step, immunofluorescent staining is performed after brief period of fixation (<1 day) using a primary antibody for the marker protein and a secondary antibody coupled to a fluorosphore. Finally, the neuron is permanently stained via the biocytin-horseradish peroxidase (HRP) reaction in which diaminobenzidine (DAB) is converted in a dark brownish precipitate. This allows high resolution morphological reconstructions of the labeled neurons (Figure 3C). It should be noted, however, that this multiple staining protocol may compromise the efficiency and quality of the biocytin-HRP staining to some extent, especially when the waiting time between fluorescence imaging and DAB processing is too long, making reconstructions of the neuronal morphology less reliable.

## Uncovering the Quantal Property of Synaptic Transmission Between Identified Cortical Neurons

As described above, postsynaptic responses in postsynaptic neurons induced by presynaptic neuronal firing fluctuate in amplitude with time; in some trials the presynaptic AP may even fail to elicit a PSP. These fluctuations have been interpreted in the framework of the quantal analysis of synaptic transmission. Quantal analysis extracts the basic functional properties of synapses from postsynaptic responses using statistical models based on some assumptions (for review, see Korn and Faber, 1991). It can give an insight into the function of synapses and identify the locus of changes in synaptic strength (Stevens, 1993). Three parameters are adopted to describe the synaptic properties: the number of release sites (*N*), the release probability (*p*), and the amplitude of postsynaptic response following a single vesicle release—the quantum (*q*). The size of postsynaptic response and its variability are determined by these quantal parameters. Presynaptic modulation is related to *p* (i.e., the release probability), while postsynaptic changes (i.e., in the number of postsynaptic receptors etc.) are related to *q*. The formation of new contacts would be related to a change in *N*. In addition, an increase in *p* from zero at existing release sites in so-called “silent” synapses could also be treated as an increase in *N*. In the past years, paired recordings in different preparations including the neocortex, hippocampus, striatum, and cerebellum have been extensively used to uncover the values for parameters *N, p*, and *q* of synaptic connections (Bekkers and Stevens, 1990; Malinow and Tsien, 1990; Larkman et al., 1991; Gulyas et al., 1993; Isaac et al., 1995; Liao et al., 1995; Scheuss et al., 2002; Silver et al., 2003; Koos et al., 2004; Biro et al., 2005; Saviane and Silver, 2006; Bremaud et al., 2007; Hardingham et al., 2010; Huang et al., 2010; Molnar et al., 2016).

**Figure 4:**
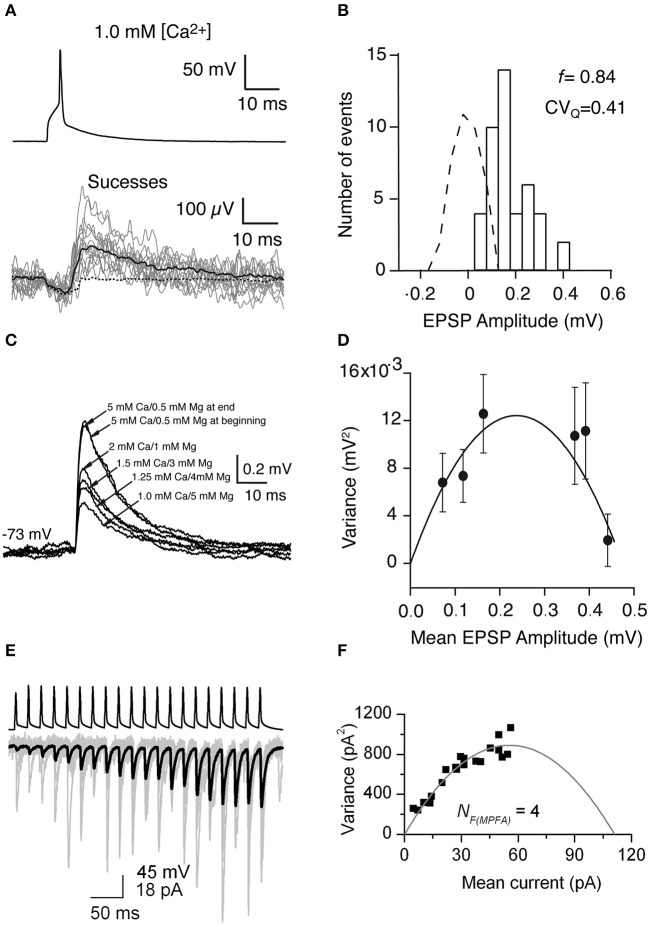
Uncovering quantal properties of synaptic transmission between identified cortical neurons. **(A)** Top, the single AP evoked by a brief suprathreshold depolarizing current pulse. Bottom, 14 individual EPSPs (gray traces), the mean of the 44 EPSP successes (black solid line), and the mean of the 279 failures (black dashed line) recorded at −72 mV in 1 mM [Ca^2+^] and 5 mM [Mg^2+^]. **(B)** Histogram of EPSP amplitude and scaled baseline noise (dashed line). For EPSP recordings from this specific synaptic connection, the failure rate (f) is 0.84 and the coefficient of variation of the quantal EPSP amplitude (*CV*_Q_) is 0.41 which was calculated from the background-subtracted variance. **(C)** Mean EPSPs recorded in different extracellular Ca^2+^ and Mg^2+^ concentrations at a postsynaptic membrane potential of −73 mV. **(D)** Relationship between the variance of the EPSP amplitude which was corrected for background variance and mean EPSP amplitude for a synaptically coupled L4-L2/3 cell pair. Each data point shows a different release probability condition. Error bars indicate the theoretical standard error in the estimate of the variance. Solid line shows the fit to a multinomial model with *q* = 0.09 mV, *N*_F_ = 5.25, and α = 19,800. **(E)** A brief train of 20 APs (top) in a presynaptic CA1 pyramidal cell evoke facilitating EPSCs in an oriens-alveus interneuron. Individual EPSCs are shown in gray and the averaged EPSC in black. **(F)** Relationship between the variance values of the postsynaptic responses which were calculated at each AP of the train and the mean current. A multinomial quantal model was fitted to the data, resulting in an *N*_F(MPFA)_ of 4, and a *q* of 24.7 pA. **(A–D)** have been adapted from Silver et al. (2003) with permission and **(E,F)** from Biro et al. (2005) with permission.

Using the frog neuromuscular junction preparation, del Castillo and Katz (Del Castillo and Katz, 1954) found that several peaks appear in the PSP amplitude histogram. Later, it has been shown that the number of peaks matched the number of anatomical synaptic contacts and the location of peaks is always multiple of that in the miniature PSP amplitude histogram, which led to postulate of the “one-site/one-vesicle” hypothesis (Del Castillo and Katz, 1954; Korn et al., 1981). However, at most synapses the PSP amplitude histogram displays no clear peaks. Therefore, more sophisticated methods have been introduced so that quantal analysis can be applied more generally. Clements and Silver developed the variance-mean (V-M) analysis of synaptic transmission, also called multiple probability fluctuation analysis, MPFA (Clements and Silver, 2000). The variance and mean are calculated from the fluctuation of PSP amplitudes in response to a presynaptic AP. A fundamental feature of this method is that it explores the fluctuation of synaptic responses at different *p* (induced by altering the extracellular Ca^2+^ concentration) (Figure 4C), therefore it can provide more information about the underlying synaptic mechanisms because of multiple points in V-M plot. Assuming that the vesicle release follows a binomial model, a plot of the variance vs. the mean of synaptic responses at different *p* displays a parabolic relationship. From the V-M plot, the values for *N, p*, and *q* can be estimated (Figure 4D). Scheuss and Neher further extended the application of the V-M analysis to the synaptic response during a train of APs (Scheuss and Neher, 2001). Instead of changing p by altering extracellular [Ca^2+^], this method allows to sample from a dynamic *p*, i.e., the PSP amplitude variation during AP train in the presynaptic neuron (Figures 4E,F). In this way, the experimental protocol is simplified because prolonged recordings are not necessary. Therefore, this approach is more readily usable.

In addition to the aforementioned univesicular release hypothesis (UVR), a multivesicular release hypothesis (MVR) has been proposed, where several vesicles are released at a single synaptic site. Recent studies in the neocortex of rodents and humans have supplied controversial evidence regarding uni- and multivesicular release. It has been reported that synaptic connections between layer 4 excitatory neurons and layer 2/3 pyramidal cells in the rat barrel cortex exhibit the UVR (Silver et al., 2003). In contrast, synaptic connections between layer 4 excitatory neurons exhibit either UVR in the primary visual cortex or MVR in the primary somatosensory cortex of mice (Huang et al., 2010). Synaptic connections between layer 5B pyramidal cells also exhibit MVR in the developing and adult somatosensory cortex of rats (Rollenhagen et al., 2018; Barros-Zulaica et al., 2019). Depending on the species, synaptic connections between pyramidal cells and interneurons exhibit either UVR in the rat neocortex or MVR in the human neocortex (Molnar et al., 2016). Therefore, transmitter release at different synaptic connections can be mediated by UVR or MVR depending on the synapse type, the cortical area and the species.

## Studying the Regulation of Synaptic Transmission by Neuromodulators

Given that synaptic transmission between individual neuron pairs is the basic unit in information processing in the brain, it is crucial to understand how synaptic transmission is dynamically regulated by neuromodulators. Neuromodulator receptors are ubiquitously distributed in the brain and can be found on both dendrites and axon terminals of excitatory and inhibitory neurons (Marder, 2012). Most neuromodulators, such as acetylcholine, norepinephrine, dopamine, serotonin etc., are synthesized by a relatively small population of neurons located in several distinct nuclei in the basal forebrain, midbrain or brainstem. These neuromodulator-releasing neurons have long-range axonal afferents that project to many cortical areas. Once released from their axon terminals, neuromodulators can diffuse over substantial distances and act on receptors remote from their release sites (a mechanism termed “volume transmission”) (Zoli et al., 1999; Agnati et al., 2010). Other neuromodulators, such as adenosine and different types of neuropeptides (e.g., VIP, Neuropeptide Y), are locally synthesized and released by neurons and/or glial cells during neuronal network activity. Synaptic transmission between synaptically coupled neurons are constantly under the influence of neuromodulators. The effect of these neuromodulators can change the function and dynamics of cortical microcircuits in a differential way because the receptor types and their distribution may differ in pre- and postsynaptic neurons. The effects of neuromodulators can be studied by bath-application of the specific neuromodulator, their agonists and antagonists. In this way, the exact concentration of applied compounds at equilibrium is known and hence pharmacological approaches, including dose-response relationships can be applied easily to dissect the molecular mechanisms of neuromodulator effects. Bath-application of neuromodulators at different concentrations might correspond to physiological concentrations of neuromodulatory release at different brain states. For example, in the neocortex, the acetylcholine concentration changes dramatically during sleep, wakefulness, arousal and sustained attention (Himmelheber et al., 2000; Teles-Grilo Ruivo et al., 2017). It is worth noting that the concentration of bath-applied agonists needs to be carefully adjusted in the physiologically meaningful range, e.g., 1–10 μM for acetylcholine. Excessive concentrations (>100 μM for acetylcholine) should be avoided in order not to distort the quantification of the synaptic effects of neuromodulators. The effects of neuromodulators can also be studied by local puff-application of the neuromodulator itself or one of its agonists/antagonists; however, with this method the actual concentration of the neuromodulator is not known. In this way transient components of the response can be detected; this is not possible when using bath-application. By combining local puff-application of neuromodulator agonists with bath-application of neuromodulator antagonists, the subtypes of neuromodulator receptors can be determined pharmacologically. Recently, optogenetic stimulation of specific types of neuromodulator afferents (e.g., cholinergic afferents from the basal forebrain) has been applied to detect synaptic responses to the endogenous release of neuromodulators (Hedrick and Waters, 2015; Urban-Ciecko et al., 2018). Below, acetylcholine and adenosine are chosen as examples to illustrate the regulation of synaptic transmission by neuromodulators.

**Figure 5:**
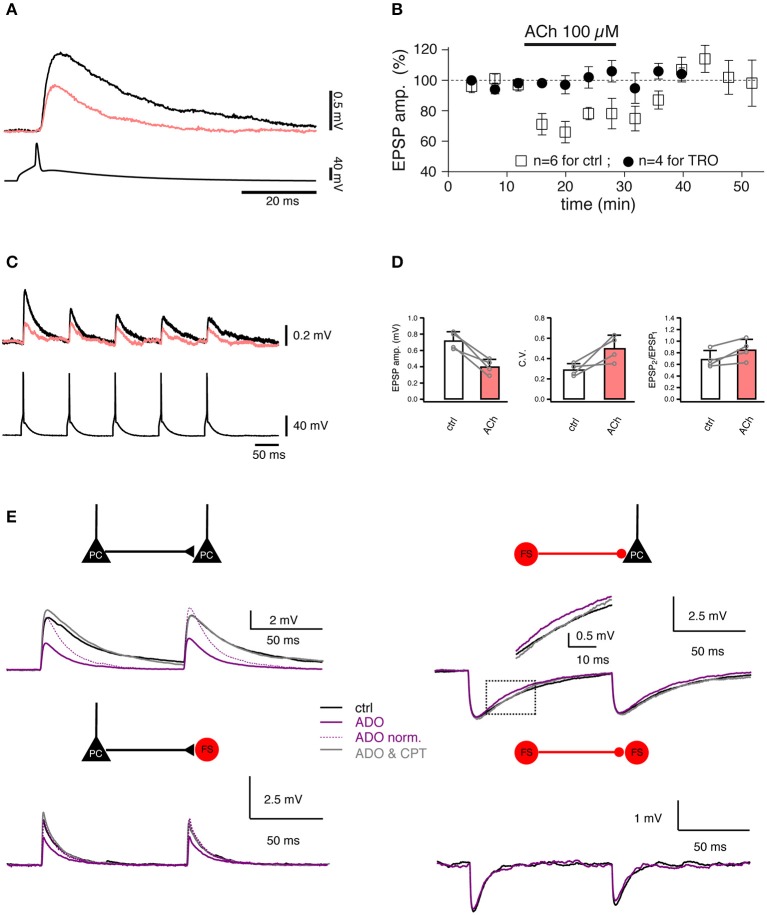
Studying the regulation of synaptic transmission by neuromodulators. **(A)** Paired recordings from a synaptic connection between two L4 spiny neurons. Bath-applied acetylcholine (ACh, 100 μM) reduces the EPSP amplitude (coral trace). **(B)** Time course of the ACh effect on the first EPSP amplitude [open boxes, control; filled circles, in the presence of 1 μM tropicamide (TRO), a selective M_4_ muscarinic acetylcholine receptor antagonist]. **(C)** Paired recordings from a synaptic connection between a L4 spiny neuron and a L2/3 pyramidal cell. A train of five APs elicited in a presynaptic L4 spiny neuron (bottom) evoked EPSPs in a postsynaptic L2/3 pyramidal cell (top) in the control condition (black) and in the presence of 100 μM ACh (coral). **(D)** Summary of the effects in L4-to-L2/3 connections (*n* = 4) in the control condition (black open box) and in the presence of 100 μM ACh (coral filled box). Left, the EPSP amplitude. Middle, the coefficient of variation (C.V.). Right, the paired-pulse ratio. Open circles are values for individual connections, connecting lines indicate the direction of change. Error bars indicate the standard deviation. **(E)** Paired recordings from synaptic connections formed between L4 spiny neurons, between L4 spiny neurons and L4 interneurons, and between L4 interneurons show that adenosine (ADO) differentially modulate the excitatory and inhibitory synaptic transmission. Overlay of average EPSPs recorded under three recording conditions: control (black), 100 μM adenosine (purple), and 100 μM adenosine plus 5 μM 8-cyclopentyltheophylline (CPT), a specific adenosine A1 receptor antagonist (gray) are shown for four connection types. **(A–D)** have been adapted from Eggermann and Feldmeyer (2009) with permission and **(E)** from Qi et al. (2017) with permission.

Acetylcholine (ACh) plays an important role in arousal, attention and vigilance. In the neocortex, ACh is released mainly from axonal boutons of neurons located in the nucleus basalis of Meynert in the basal forebrain. Cholinergic afferent terminals are distributed at high density throughout the cortical layers (Kalmbach et al., 2012). It has been proposed that most of the intra-cortical ACh is not released at synaptic contacts but rather diffusely into the extracellular space, i.e., by volume transmission. However, some evidence suggests that phasic release exists ubiquitously in the cortical cholinergic system (Sarter et al., 2009). The effects of ACh in the neocortex are mediated by two types of ACh receptors, the G-protein-coupled muscarinic AChRs (mAChRs) and the nicotinic AChR ion channels (nAChRs). It has been shown that ACh affects excitatory synaptic transmission by causing either a reduction or an increase in the release probability. An ACh-induced reduction in release probability has been shown through paired recordings of excitatory L4-L4 (Figures 5A,B) and L4-L2/3 (Figures 5C,D) synaptic connections in the rat barrel cortex (Eggermann and Feldmeyer, 2009) which exhibited a decreased EPSP amplitude and increased failure rate, variability and PPR. M_4_ mAChRs located in presynaptic L4 axonal terminals caused the suppression of synaptic release probably by decreasing the open probability of presynaptic Ca^2+^ channels. Such a suppressive effect of ACh was also found in excitatory connections established by L2/3 and L5 pyramidal neurons (Levy et al., 2006, 2008). In layer 6, the ACh effect on synaptic transmission depends on the presynaptic neuron type: ACh decreases the synaptic release probability of L6 cortico-cortical pyramidal neurons to other excitatory and inhibitory neurons via activating the presynaptically located M_4_ mAChRs. In contrast, ACh enhances the synaptic transmission originating from L6A cortico-thalamic pyramidal neurons via activating the α_4_/β_2_ nAChRs located at presynaptic axonal terminals (Yang et al., 2019). A similar nicotinic enhancement effect of ACh was found both *in vitro* and *in vivo* at synaptic connections between L2 pyramidal neurons and somatostatin-expressing interneurons (Urban-Ciecko et al., 2018).

In contrast to ACh, adenosine is an endogenous neuromodulator which is generated during high neuronal activity, e.g., by the intra- and extracellular metabolism of adenosine triphosphate. Adenosine has been suggested to play an important role in the sleep homeostasis (Porkka-Heiskanen et al., 1997, 2000). Recently, the effect of adenosine on synaptic transmission has been assessed using paired recordings (Kerr et al., 2013; Qi et al., 2017). Adenosine induces a suppression of the neurotransmitter release probability at intralaminar L2/3, L4, and L5 and translaminar L4-L2/3 excitatory connections. The adenosine effect is most likely mediated by A_1_ adenosine receptors located in presynaptic axonal terminals; they induce a reduction in the open probability of presynaptic Ca^2+^ channels involved in triggering the release of neurotransmitters. This effect is already apparent at low endogenous concentrations of adenosine (~1 μM) which are tonically released (Qi et al., 2017). In contrast, adenosine has a much smaller effect on inhibitory synaptic transmission onto excitatory neurons: here, only the IPSP time course is altered due to activation of postsynaptically located A_1_ adenosine receptors. There is no effect on inhibitory synaptic transmission onto interneurons (Figure 5E).

In addition to ACh and adenosine, a synapse type-dependent neuromodulation has also been found for other neuromodulators such as dopamine. Paired recordings from pyramidal cells and interneurons in ferret prefrontal cortex showed that dopamine depresses excitatory transmission between two pyramidal cells through D_1_ receptor actions at a presynaptic site (Gao et al., 2001) but has no effect on excitatory transmission between pyramidal cells and fast-spiking (FS) interneurons (Gao and Goldman-Rakic, 2003). In addition, dopamine differentially modulates inhibition of pyramidal cells from FS vs. non-FS interneurons. Dopamine decreases release of GABA onto pyramidal cells through effects on presynaptic D_1_ receptors on axonal terminals of FS interneurons, whereas inhibition from non-FS interneurons onto pyramidal cells is enhanced, presumably owing to a postsynaptic effect (Gao et al., 2003). Similarly, differential modulatory effects of dopamine on different types of synaptic transmission in the medial prefrontal cortex (Dembrow et al., 2010; Dembrow and Johnston, 2014) and neostriatum (Tecuapetla et al., 2007, 2009) have also been found. In summary, the effect of neuromodulators on synaptic transmission depends on the synapse type which is determined by both presynaptic and postsynaptic neuronal identities.

## Outlook

Paired recordings from synaptically coupled excitatory and/or inhibitory neurons are a powerful technique to investigate the structure-function relationship of synaptic microcircuits at the subcellular, cellular, and network level. It allows the simultaneous electrophysiological, morphological and/or molecular analysis of both the pre- and postsynaptic neurons in synaptic connections. This is as yet difficult if not impossible for other techniques using extracellular (electrical or optical) stimulation of presynaptic neurons, see e.g., Crochet et al. (2005) and Pala and Petersen (2018). In addition, long-time stable paired recordings permit an in-depth characterization of a defined unitary synaptic connection using, e.g., the quantal analysis. Furthermore, agonist and/or antagonist can be applied readily to neurons in slice preparations (and even spatially focussed), which allows studying the effects of neuromodulators on the synaptic transmission. However, to appreciate the insight obtained from paired recordings in brain slices, one needs to be aware of several shortcomings.

A major disadvantage of slice preparations is the often substantial truncation of axonal branches so that only parts of the axon are reserved in the 300–400 μm-thick brain slice. For some pyramidal cell types, the degree of truncation could be up to 90% when taking into account projections to other cortical or subcortical areas (Stepanyants et al., 2009; Narayanan et al., 2015). Therefore, the slice preparation is not suited for the study of synaptic connections between neurons whose cell bodies are more than >300 μm in the lateral direction. For studying synaptic connections between neurons with inter-soma distances >500 μm within the same column, e.g., translaminar L2/3-to-L5 or L4-to-L6 connections (Reyes and Sakmann, 1999; Qi and Feldmeyer, 2016), paired recordings in the slice preparation is still usable when the slicing procedure is optimized. However, *local* axonal projections, in particular those of interneurons are generally recovered with a relatively low degree of truncation (~10% or less) (Koelbl et al., 2015; Emmenegger et al., 2018) because of their limited horizontal and vertically projections (see Movie S1). Synaptic connections involving these neuron types can therefore be characterized with high accuracy and reliability and their connectivity estimates are largely correct. Except for these local synaptic connections, absolute values for connectivity ratios between two neuron types obtained in slice preparations are highly questionable, in particular for those with large inter-somatic distances such as translaminar or non-local intralaminar synaptic connections. This problem is even more prominent when slicing procedures have not been optimized for a given synaptic connection at a defined developmental stage. Another problem for connectivity estimates is that distal synaptic contacts, e.g., those on the apical tuft dendrites of pyramidal neurons, may escape detection (Williams and Stuart, 2002, 2003). When recorded at the soma the amplitude of their synaptic response is very small and therefore likely to be obscured by electrical noise. However, this type of problem is not confined to the paired recording approach but could also arise in other techniques adopted to study the synaptic connectivity.

In recent years light-induced activation of neurons by photo-release of caged glutamate (Callaway and Katz, 1993) or by activation of channelrhodopsin-2 channels expressed in different neuronal compartments, e.g., soma, dendrites (Boyden et al., 2005), or axonal terminals (Petreanu et al., 2007) has been used to investigate neuronal microcircuits on a larger scale. However, it is so far not possible to identify the detailed structural properties of presynaptic neurons with these optical approaches. Furthermore, the number and location of synaptic contacts for a synaptic connection cannot be identified. Paired recordings, however, allow a detailed characterization of both pre- and postsynaptic neurons and their synaptic contacts in a synaptic connection. This is of paramount importance because many studies have demonstrated that both GABAergic interneurons and glutamatergic excitatory neurons in the neocortex are highly diverse with respect to their morphologies and synaptic properties. Therefore, the identification of both pre- and postsynaptic neurons is necessary for a deep characterization of a synaptic connection.

To enhance the success rate of recording synaptic connections in local neuronal microcircuits, the number of simultaneously recorded neurons (*n*) has been increased from dual (2), triple (3), quadruple (4), octuple (8) up to 12 (Thomson et al., 2002; Song et al., 2005; Kampa et al., 2006; Brown and Hestrin, 2009; Lefort et al., 2009; Yu et al., 2009; Ko et al., 2011; Perin et al., 2011; Rieubland et al., 2014; Jiang et al., 2015; Guzman et al., 2016; Peng et al., 2017; Hemberger et al., 2019). Multiple (*n* > 2) recordings may yield more synaptic connections because the number of potential synaptic connections (*m*) established between n neurons increases steeply with increasing *n*: *m* = *n* × (n−1). However, multiple recordings especially when *n* > 4 have several shortcomings compared to paired recordings. First, the mechanical stability will decrease the more electrodes are placed together in the recordings chamber while the electrical noise of the recording will increase substantially because of capacitive coupling (electrical “cross-talk”) in multichannel electrophysiology experiments. This is particularly problematic when the two recorded signals are not of similar amplitude as is the case in paired recordings (presynaptic AP vs. small postsynaptic response) (Nelson et al., 2017). It is likely to decrease the probability of successful, high resolution recordings from a large number of neurons. In addition, the quality of the measured signals (i.e., the signal to-noise ratio of the recordings) will also deteriorate so that the detection of small PSPs (10–20 μV) is severely compromised (Seeman et al., 2018). Furthermore, the time for recording from an individual synaptic connections will be relatively short, i.e., the characterization of this connection limited because of the restricted overall total recording time for all possible synaptic connections. Therefore, a detailed functional characterization of the properties of unitary PSPs (e.g., quantal analysis) is very difficult. Moreover, when biocyin is added to pipettes during multiple recordings, many neurons will be stained in the same slice after the histochemical processing, which makes a reliable and complete reconstruction of neuronal morphology (including both the dendritic and axonal branches) extremely complicated if not impossible, especially when more than two interneurons with a dense axonal plexus are involved. Finally, the estimate of connectivity ratios for all connection types using multiple recordings in the same slice preparation is likely to be unreliable in particular for translaminar or non-local intralaminar synaptic connections because the slicing procedure is optimal only for a few specific connection types (mainly the local ones) but not for the majority. This problem could be overcome in paired recordings through optimizing the slicing procedure for *specific* types of synaptic connections. Despite of aforementioned shortcomings that exist so far, multiple recordings show great promise for future high-throughput analysis of cortical microcircuits in rodent and more precious human brains (Peng et al., 2019).

Not only cortical inhibitory but also excitatory neurons show a high diversity (Zeng and Sanes, 2017). To directly target specific neuronal subpopulations, paired recordings have been conducted in acute brain slices from transgenic animals where one specific or several populations of neurons are labeled by fluorescent groups (e.g., GFP, YFP, tdTomato etc.) as in transgenic, knock-in animals or via viral infection (Pfeffer et al., 2013; Seeman et al., 2018). Paired recordings can be combined easily with other cutting-edge techniques, such as optogenetics, Ca^2+^ imaging, activity-dependent immediate early gene expression and pseudorabies virus retrograde tracing etc. (Wickersham et al., 2007; Yassin et al., 2010; Ko et al., 2011; Jouhanneau et al., 2014; Lee et al., 2014; Cossell et al., 2015; Morgenstern et al., 2016). More recently, the paired recording approach has also been adopted to record from synaptically coupled neurons in the intact brain of anesthetized mice (Jouhanneau et al., 2015, 2018). Paired recordings have also been used to investigate the functional and structural properties of synapses in surgically dissected human brain slices (Molnar et al., 2008; Testa-Silva et al., 2010, 2014; Boldog et al., 2018; Seeman et al., 2018). For human tissue, paired recording in slices is still the only method of choice to study the functional neuronal microcircuits in preparations from human brains. Therefore, paired recordings will remain an important approach for studying neuronal microcircuits in different brain regions and species.

## Author Contributions

GQ generated the figures. GQ and DF wrote the manuscript with comments and suggestions from DY and CD.

### Conflict of Interest

The authors declare that the research was conducted in the absence of any commercial or financial relationships that could be construed as a potential conflict of interest.

## Supplementary Material

Movie S1 Rotation of a 3D morphologically reconstructed parvalbumin+ (PV+), fast-spiking interneuron in layer 4 of rat barrel cortex. The somatodendritic compartment is shown in red and the axon in cyan. Borders between different layers are also shown.
